# Supplementary material for: A Large-Scale Validation of an Improved Embryo-Rescue Protocol for the Obtainment of New Table-Grape Seedless Genotypes
Source: Plants (Basel). 2023 Oct 3;12(19):3469. doi: 10.3390/plants12193469 (PMC10575281; doi:10.3390/plants12193469)
Supplement: Supplementary file 1 [file plants-12-03469-s001.zip › plants-2609636-supplementary.pdf]

**Supplementary material Table S1**: Numbers of ovules per berry and percentage of developed embryos in crosses between different grapevine genotypes at different sampling time.

| Ripening time* | Cross (♀ × ♂) | Features** | I | II | III | IV |
|---|---|---|---|---|---|---|
| M | TS01 × TS04 | Ov/Be | 2.3 | 2.0 | n.a. | 2.5 |
| | | %E | 4.0 | 4.1 | n.a. | 4.2 |
| M | TS02 × TS04 | Ov/Be | 1.9 | 1.6 | n.a. | 1.8 |
| | | %E | 15.7 | 20.2 | n.a. | 10.8 |
| M | TS11 × TS24 | Ov/Be | n.a. | 2.3 | 2.1 | n.a. |
| | | %E | n.a. | 7.6 | 13.7 | n.a. |
| M | TS12 × TS24 | Ov/Be | 1.8 | 1.5 | n.a. | n.a. |
| | | %E | 3.6 | 10.0 | n.a. | n.a. |
| M | TS13 × TS24 | Ov/Be | 2.0 | 2.1 | n.a. | n.a. |
| | | %E | 11.6 | 15.0 | n.a. | n.a. |
| M | TS13 × TS24 | Ov/Be | 2.0 | 2.0 | n.a. | n.a. |
| | | %E | 10.6 | 12.2 | n.a. | n.a. |
| M | TS13 × TS27 | Ov/Be | n.a. | 1.7 | 2.1 | n.a. |
| | | %E | n.a. | 4.2 | 3.4 | n.a. |
| M | TS16 × TS27 | Ov/Be | 1.9 | 2.0 | n.a. | 1.9 |
| | | %E | 24.7 | 29.0 | n.a. | 46.9 |
| M | TS27 × TS03 | Ov/Be | n.a. | 3.5 | 3.4 | 2.9 |
| | | %E | n.a. | 6.5 | 9.5 | 18.2 |
| M | TS27 × TS21 | Ov/Be | 2.3 | 2.3 | n.a. | n.a. |
| | | %E | 8.0 | 11.6 | n.a. | n.a. |
| M | TS27 × TS32 | Ov/Be | n.a. | 3.0 | 3.4 | 3.3 |
| | | %E | n.a. | 12.1 | 11.8 | 7.5 |
| M | TS28 × TS04 | Ov/Be | 1.2 | n.a. | n.a. | 1.2 |
| | | %E | 15.6 | n.a. | n.a. | 20.0 |
| M | TS28 × TS13 | Ov/Be | 1.1 | n.a. | n.a. | 1.0 |
| | | %E | 11.5 | n.a. | n.a. | 17.1 |
| ML | TS04 × TS24 | Ov/Be | 2.3 | 2.6 | 1.6 | n.a. |
| | | %E | 0.8 | 0.9 | 1.0 | n.a. |
| ML | TS23 × TS24 | Ov/Be | n.a. | 2.5 | 2.4 | n.a. |
| | | %E | n.a. | 14.8 | 20.1 | n.a. |
| ML | TS23 × TS28 | Ov/Be | n.a. | 2.5 | 1.5 | n.a. |
| | | %E | n.a. | 20.6 | 23.3 | n.a. |
| ML | TS33 × TS19 | Ov/Be | n.a. | 2.6 | n.a. | 2.1 |
| | | %E | n.a. | 6.7 | n.a. | 18.8 |
| ML | TS33 × TS28 | Ov/Be | n.a. | 2.2 | n.a. | 2.5 |
| | | %E | n.a. | 13.4 | n.a. | 27.6 |
| ML | TS37 × TS19 | Ov/Be | n.a. | 2.3 | n.a | 2.2 |
| | | %E | n.a. | 9.0 | n.a. | 14.7 |
| L | TS05 × TS27 | Ov/Be | n.a. | 2.2 | 1.7 | n.a. |
| | | %E | n.a. | 15.4 | 22.4 | n.a. |
| L | TS09 × TS04 | Ov/Be | 2.5 | n.a. | 2.2 | n.a. |
| | | %E | 7.0 | n.a. | 5.6 | n.a. |
| L | TS09 × TS10 | Ov/Be | n.a. | 2.5 | n.a. | 1.9 |
| | | %E | n.a. | 9.6 | n.a. | 1.6 |
| L | TS09 × TS13 | Ov/Be | n.a. | 2.3 | n.a. | 2.4 |
| | | %E | n.a. | 16.1 | n.a. | 22.0 |

| | | | | | | |
|---|---|---|---|---|---|---|
| L | TS25 × TS13 | Ov/Be | n.a. | 2.1 | n.a. | 1.4 |
| | | %E | n.a. | 0.9 | n.a. | 3.9 |
| L | TS25 × TS18 | Ov/Be | n.a. | 1.9 | 2.2 | n.a. |
| | | %E | n.a. | 1.6 | 1.2 | n.a. |
| L | TS25 × TS39 | Ov/Be | n.a. | 1.2 | n.a. | 1.5 |
| | | %E | n.a. | 1.9 | n.a. | 2.6 |

*: M: Middle; ML: Middle-Late; L: Late

**:I: 43-47 DAP harvest; II: 48-52 DAP harvest; III: 53-57 DAP harvest; IV: 58-62 DAP harvest.

**Supplementary material Table S2.** Composition of medium used for ovules establishment.

| Components | Quantity |
|---|---|
| **Nitsch J.P Macroelements** [45] | |
| Ammonium nitrate ($NH_4NO_3$) | 0.720 g/l |
| Calcium chloride ($CaCl_22H_2O$) | 0.166 g/l |
| Magnesium sulphate heptahydrate ($MgSO_47H_2O$) | 0.185 g/l |
| Potassium nitrate ($KNO_3$) | 0.950 g/l |
| Potassium phosphate monobasic ($KH_2PO_4$) | 0.068 g/l |
| **Nitsch&Nitsch's Microelements** [45] | |
| Manganese sulphate monohydrate ($MnSO_4H_2O$) | 25.0 mg/l |
| Copper sulphate pentahydrate ($CuSO_4$ $5H_2O$) | 0.025mg/l |
| Zinc sulphate heptahydrate ($ZnSO_4$ $7H_2O$) | 10.0 mg/l |
| Boric acid ($H_3BO_3$) | 10.0 mg/l |
| Sodium molybdate dihydrate ($Na_2MoO_4$ $2H_2O$) | 0.25 mg/l |
| **Iron chelate** | |
| Iron sulfate ($FeSO_4$) | 50.00 mg/l |
| Sodium EDTA (NaEDTA) | 50.50 mg/l |
| **Hormones** | |
| GA3 | 2.00 mg/l |
| IAA | 3.00 mg/l |
| **Nitsch&Nitsch's vitamins (1969)** | |
| Myo-Inositol | 100.00 mg/l |
| Thiamine hydrochloride | 0.5 mg/l |
| Pyridoxine | 0.5 mg/l |
| Glycine | 2.0 mg/l |
| Nicotinic acid | 5.0 mg/l |
| Biotin | 0.05 mg/l |
| Folic acid | 0.50 mg/l |
| **Agar (Duchefa Biochemie phyto AGAR)** | 6.50 g/l |
| **Saccharose** | 20.00 g/l |
| **Activated Charcoal** | 2.00 g/l |
| **pH 5.8** | |

**Supplementary material Table S3.** Composition of the medium used for embryo growth.

| Components | Quantity |
|---|---|
| **Quoirin Leipovre Macroelements (1977)** [52] | |
| Potassium nitrate ($KNO_3$) | 4.200 g/l |
| Ammonium nitrate ($NH_4NO_3$) | 0.800 g/l |
| Magnesium sulphate heptahydrate ($MgSO_47H_2O$) | 0.720 g/l |
| Potassium phosphate monobasic ($KH_2PO_4$) | 0.540 g/l |
| Calcium nitrate tetrahydrate ($Ca(NO_3)_2\ 4H_2O$) | 0.068 g/l |
| **Murashige&Skoog Microelements (1962**) [53] | |
| Manganese sulphate monohydrate ($MnSO_4H_2O$) | 16.9 mg/l |
| Zinc sulphate heptahydrate ($ZnSO_4\ 7H_2O$) | 8.6 mg /l |
| Boric acid ($H_3BO_3$) | 6.2 mg/l |
| Copper sulphate pentahydrate ($CuSO_4\ 5H_2O$) | 0.025 mg/l |
| Cobalt chloride ($CoCl_2\ 6H_2O$) | 0.025 mg/l |
| Sodium molybdate dihydrate ($Na_2MoO_4\ 2H_2O$) | 0.25 mg/l |
| Potassium iodide (KI) | 0.83 mg/l |
| **Iron chelate** | |
| Iron sulfate ($FeSO_4$) | 50.0 mg/l |
| Sodium (EDTA NaEDTA) | 55.0 mg/l |
| **Vitamins Z4** | |
| Inositol | 10.00 g/l |
| Thiamine | 0.10 g/l |
| Pyridoxine | 0.05 g/l |
| Glycine | 0.20 g/l |
| Nicotinic acid | 0.05 g/l |
| Ascorbic acid | 1.00 g/l |
| Citric acid | 1.00 g/l |
| **Agar (Duchefa Biochemie phyto AGAR)** | 6.50 g/l |
| **Saccharose** | 20.00 g/l |
| **pH 5.7-5.8** | |
